# Ten quick tips for editing Wikidata

**DOI:** 10.1371/journal.pcbi.1011235

**Published:** 2023-07-20

**Authors:** Thomas Shafee, Daniel Mietchen, Tiago Lubiana, Dariusz Jemielniak, Andra Waagmeester

**Affiliations:** 1 Swinburne University of Technology, Melbourne, Australia; 2 Ronin Institute, Montclair, New Jersey, United States of America; 3 Institute for Globally Distributed Open Research and Education (IGDORE), Gothenburg, Sweden; 4 Leibniz Institute for Freshwater Ecology and Inland Fisheries (IGB), Berlin, Germany; 5 FIZ Karlsruhe–Leibniz Institute for Information Infrastructure, Berlin, Germany; 6 University of São Paulo, São Paulo, Brazil; 7 Kozminski University, Warsaw, Poland; 8 Micelio, Ekeren, Belgium; McGill University, CANADA

This is a *PLOS Computational Biology* Software paper.

## Introduction

This article acts as a successor to the 10 simple rules for editing Wikipedia from a decade ago [1]. It addresses Wikipedia’s machine-readable cousin: Wikidata—a project potentially even more relevant from the point of view of Computational Biology.

Wikidata is a free collaborative knowledgebase [2] providing structured data to every Wikipedia page and beyond. It relies on the same peer production principle as Wikipedia: anyone can contribute. Open, collaborative models often surprise in how productively they work in practice, given how unlikely they might be expected to work in theory. Nevertheless, they can still be met with a lot of resistance and suspicion in academic circles [3,4].

**Fig 1:**
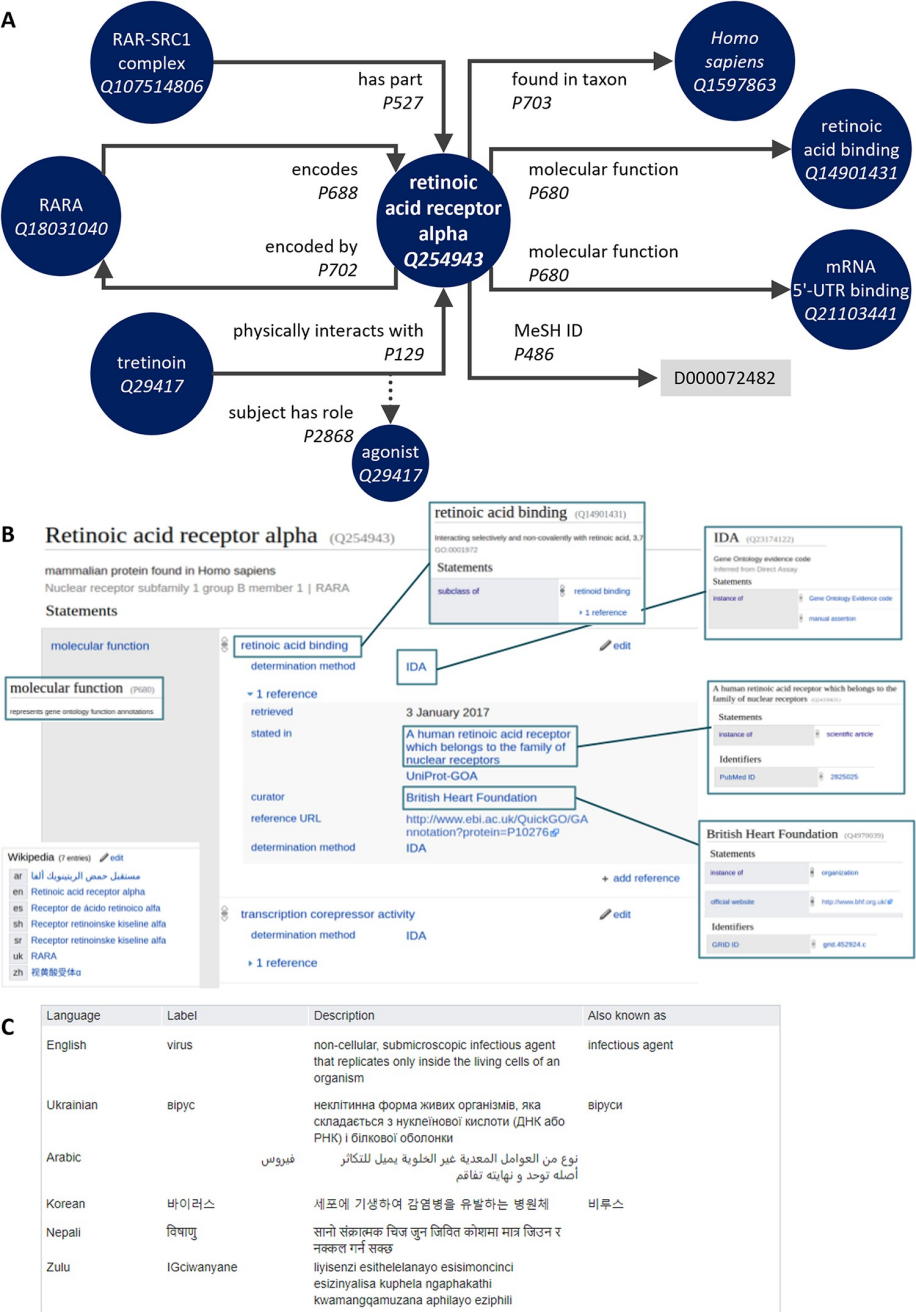
Structure of an example Wikidata item. Wikidata items are linked to one another and to outside databases via properties that describe the relationships between them. (**A**) Some example links to and from the item *human retinoic acid receptor alpha (Q254943)*. Items can have outgoing links; e.g., to the concept of a *protein (Q8054)*, incoming links; e.g. from the *human RAR–SRC1 complex (Q107514806)*, or both; e.g., to and from the *human RARA gene (Q18031040)*. There can be multiple links out with the same property (e.g., multiple molecular functions) and links out to external websites and identifiers; e.g., it has the *MeSH ID (P486)* of D011506. The links formed by properties can be further annotated with qualifiers; e.g., its *physical interaction with (P129) tretinoin (Q29417)* is with the *role of (P2868)* being an *agonist (Q389934)*. Now imagine this for a hundred million node items and many billions of property edges. (**B**) The human–readable interface for this item is organised into the label, description, and aliases, followed by a list of statements with their qualifications and references, with a final section listing any Wikipedia (and other wikimedia) pages for the item. (**C**) Example labels, descriptions, and aliases for *virus (Q808)* from the 410 currently supported languages. *These screenshots contain only text and data released under a CC0 licence*.

Since its launch in 2012, Wikidata has rapidly grown into a cross-disciplinary open knowledgebase with items ranging from genes to cell types to researchers [2,5–7]. It has wide-ranging applications, such as validating statistical information about disease outbreaks [8], aligning resources on human coronaviruses [9], or assessing biodiversity [10,11]. It can be thought of as a vast network graph (Fig 1A), wherein the items act as nodes (now over 100 million) linked to one another by over a billion statements, and further linked out to the wider web by many billions more. We’ll link to example Wikidata items and properties by using italics throughout the text as we refer to them (Fig 1).

The online interface makes the items themselves somewhat human-readable (Fig 1B), but their structured nature makes it possible to query and combine the information in ways that can’t be achieved for information sources written entirely in prose. This versatility makes its applications in computational biology, arguably, even more universal and flexible than just relying on Wikipedia alone [12]. Queries on Wikidata can vary from which gene variants predict a positive prognosis in colorectal cancer to taxa by number of streets in the Netherlands that bear their name. We’ll try to use examples relevant to computational biology, but bear in mind that the same can be done with almost everything from a map of mediaeval witch executions in Scotland to emergency phone numbers by population using them to paintings depicting frogs.

Since it’s under a CC0 copyright waiver, Wikidata’s structured content is essentially released into the public domain to be used on other projects [13]. You’ll probably have already seen its structured data at the top of search engine results but it’s also used behind the scenes on thousands of sites, becoming the backbone infrastructure for using, sharing, and collaboratively curating structured reference knowledge.

### Tip 1: Learn by doing

If you’re thinking of editing Wikidata, you can start right away, perhaps by exploring and experimenting with one of its sandbox items like Q4115189, or by taking some of the introductory tours. While it is possible to edit without an account, it is best to register one. Wikidata uses the same user account as Wikipedia or Wikimedia Commons. This enables you to build a reputation within the editor community as you contribute, makes it easier for other editors to contact and collaborate with you, and will enable you to use some additional tools (see Tip 9). Paradoxically, it can also protect your anonymity better: you edit under a username of your choice instead of your edits being tagged with your IP address. Once you’ve created your account, it’s useful to click on your username in the top right of the screen to add some basic information to your userpage—particularly your topics of interest and your areas of expertise. It is increasingly common, although not required, for researchers on Wikidata to also link out to their real-world identity (faculty profile, professional social media, personal website, etc.) or simply to the Wikidata entry about them.

Whereas Wikipedia strictly prohibits editing a page about yourself (if you have one), in Wikidata, it is acceptable to add uncontroversial statements to the Wikidata item about you if you can reference them to publicly available sources (see Tip 7). It can therefore be useful to search for yourself in Wikidata and add statements, for example, your *ORCID (P496)*, *Github account (P2037)*, or *Wikimedia username (P4174)*. Also note that while it is technically possible to add phone numbers or email addresses, be extremely cautious about adding any information—to any item—that may violate privacy (the policy about living people provides guidance here).

### Tip 2: Think of knowledge as structured statements

Information in Wikidata is organised into statements. A basic statement is a triple containing a subject, a predicate, and an object. Although the subject of a statement is always a Wikidata item, the object can be either another Wikidata entity or another data type such as strings, URLs, quantities, or external identifiers. For example, *Human retinoic acid receptor alpha (Q254943)* has the *molecular function (P680)* of *retinoic acid binding (Q14901431)* (Fig 1). The identifiers beginning with Q are items and indicate objects, concepts, or events. Identifiers beginning with P are the properties that define relationships.

This model of statements is common to linked data repositories aligned to the Semantic Web [14–16], and Wikidata extends it with qualifiers and references that enable capturing specific detail and provenance (see Tip 7). For example, the statement *Retinoic acid receptor alpha (Q254943) physically interacts with (P129) tretinoin (Q29417)*, *with the role (P2868) of agonist (Q389934)* cites as a reference that it is *stated in (P248)* the *IUPHAR/BPS database (Q17091219)*.

Besides Ps and Qs, some other identifiers with a leading letter are important in the Wikidata ecosystem. For example, identifiers starting with Ls are for lexemes that indicate linguistic properties of a word or phrase, e.g., the Swedish noun *“modell” (L47542)* has multiple meanings, only one of which is a simplified representation of reality *(Q1979154)*. Similarly, Wikidata identifiers starting with E are for entity schemas, which are particularly useful for defining and validating items (see Tip 9).

Wikidata is based on the knowledge graph management software Wikibase. Since the software is open-source, it is also used in a range of other specialist applications to host data as structured statements. Learning this way of thinking about information therefore enables participation beyond Wikidata. The main other example within the Wikimedia ecosystem is annotation of the Wikimedia Commons media-sharing platform. It is also being implemented in projects outside of Wikimedia that range from ontologies for botanical collections [17], a semantic map of the trade of enslaved people [18], or general research data management applications [19].

### Tip 3: Take a look at what’s already there

The main reason to have data in a multidisciplinary knowledgebase is to be able to extract and combine it in interesting ways. It is possible to search for and view items individually via the user interface on the web or browse geographically nearby items, but a more powerful counterpart to this is to explore the data using database queries. Wikidata can be queried using the SPARQL language via tools such as the Wikidata Query Service. It is worth noting that queries are organised around semantic concepts rather than simple keyword text strings, so searching for “diseases associated with human pancreatic beta cells via markers” is essentially asking “find items listed as gene markers of human beta cells; for those genes, find diseases associated with them; count how often each gene occurs.”

**Fig 2:**
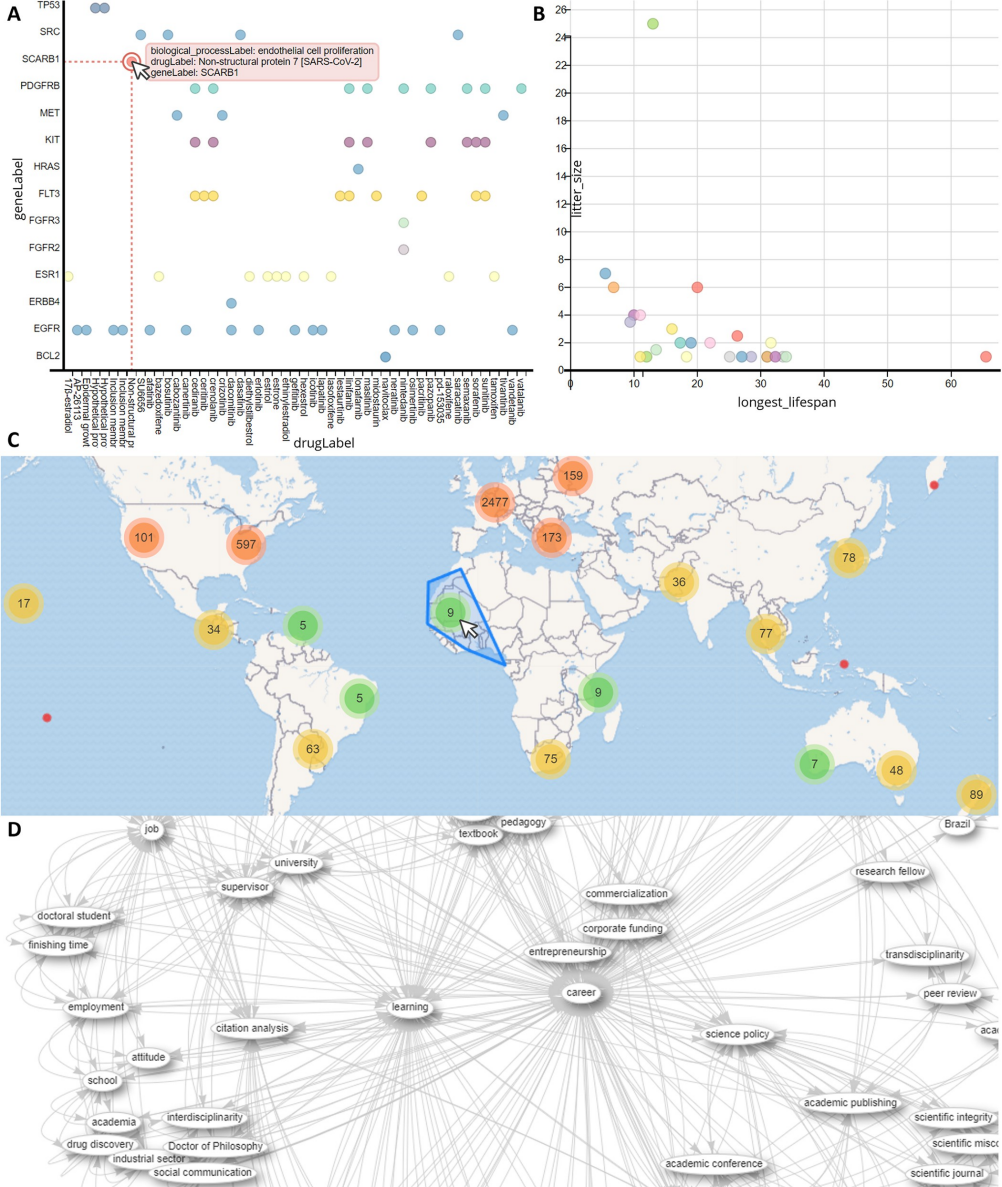
Example visualisations generated using the Wikidata Query Service. (**A**) Which drugs target genes involved in cell proliferation. (**B**) Litter size versus lifespan for endangered species. (**C**) Birthplaces of people after whom taxa are named. (**D**) Co–occurance of main topics of PLOS “Simple Rules” and “Quick Tips” articles (cropped to subset around learning and career). *The map image is produced using base map and data from* *OpenStreetMap* *and the OpenStreetMap Foundation*.

The Wikidata Query Service also has several inbuilt lightweight visualisation options. The simplest is probably scatterplots of categorical (Fig 2A) and continuous (Fig 2B) data. For geographical data, it is possible to overlay coordinates over a map (Fig 2C). In the self-referential tradition of the Ten Simple Rules series [20], looking at the subset of the Wikidata network showing co-occurrence of main subjects in the “Simple Rules” and “Quick Tips” articles series illustrates the main clusters around the themes of career, learning, and software (Fig 2D). These visualisations are usually best viewed interactively, so links to the SPARQL queries are included in the figure legend. For those not experienced with SQL queries, you can request help for building queries (see Tip 9).

Some of these example queries are highly specialist, whereas others take advantage of Wikidata’s interdisciplinarity to show things that are difficult to answer with specialist databases, such as combining biological, historical, and geographic data to illustrate a sociological phenomenon of taxon naming bias (Fig 2C).

Some sites, such as Scholia (an open-source alternative to the likes of Google Scholar), are built entirely from Wikidata query visualisations [21], for example, this summary of publications about human astrocytes. The Wikidata Query Service also provides a code section from which snippets can be copied into different programming tools for more in-depth analysis and visualisation. Many of those tools are also able to dynamically read from and write to Wikidata so that items can be kept dynamically up-to-date and integrated with other programming pipelines (see Tip 9).

### Tip 4: Join a community

Wikidata’s community portals are available in multiple languages (Box 1) and have broad introductory help and training; however, the majority of its help comes from its community of users, who can be contacted individually or via several rapid-response locations like general project chat (discussions are archived after about a week). There is also a request-a-query page to ask for assistance in creating or refining SPARQL queries. Wikiprojects are self-organised communities of practice consisting of volunteers and their bots, focused on items in a specific topic, their structure, and typology, and are typically highly communicative and supportive to each other [22]. Some have relatively broad scope (e.g., Molecular Biology, Taxonomy, Medicine, Source MetaData) and others are more narrow (e.g., COVID-19, Haplogroups)—see the full directory here. These projects have discussion pages as well as exemplar items that can help you align newly added content with best practice (see Tip 6). In case of more serious problems or dispute resolution, consulting with current admins may be useful, too.

Additionally, it is possible to join Wikidata communities outside the Wikidata platform. Wikidata’s contributors are generally keen to help and collaborate with anyone interested in the platform, so consider also reaching out to researchers who’ve used Wikidata. You can join in-person events organised by the Wikimedia affiliates in most regions. Since multiple groups might exist for any given topic, so once you have found one that resonates with your interests, keep an eye out for others exploring other facets of the same topic. Finally, there are active Wikidata communities on social media platforms such as Mastodon, Telegram, Twitter, or Facebook.

**Box 1. Community portal languages**

Afrikaans, Bahasa Indonesia, Bahasa Melayu, Basa Bali, British English, Bân–lâm–gú, Chi–Chewa, Cymraeg, Deutsch, English, Esperanto, Frysk, Gaeilge, Gagana Samoa, Ghanaian Pidgin, Hausa, Ido, Igbo, Ilokano, Jawa, Kreyòl ayisyen, Lingua Franca Nova, Lëtzebuergesch, Malagasy, Minangkabau, Mirandés, Māori, Nederlands, Ripoarisch, Scots, Sesotho sa Leboa, Simple English, Sunda, Tiếng Việt, Tyap, Türkçe, Volapük, Zazaki, asturianu, azərbaycanca, bosanski, brezhoneg, català, dansk, dolnoserbski, eesti, emiliàn e rumagnòl, español, euskara, eʋegbe, français, føroyskt, galego, hornjoserbsce, hrvatski, interlingua, isiXhosa, italiano, kurdî, latviešu, lietuvių, magyar, norsk bokmål, occitan, polski, português, português do Brasil, română, shqip, slovenčina, suomi, svenska, tarandíne, tatarça, toki pona, vèneto, íslenska, čeština, ślůnski, Ελληνικά, башҡортса, беларуская, беларуская (тарашкевіца), български, македонски, монгол, русский, српски / srpski, татарча / tatarça, тоҷикӣ, українська, қазақша, հայերեն, ייִדיש, עברית, ئۇيغۇرچە, ئۇيغۇرچە / Uyghurche, اردو, العربية, بهاس ملايو, تۆرکجه, سرائیکی, سنڌي, فارسی, مصرى, پښتو, ߒߞߏ, अंगिका, अवधी, भोजपुरी, मगही, मराठी, हिन्दी, অসমীয়া, বাংলা, ਪੰਜਾਬੀ, ગુજરાતી, தமிழ், తెలుగు, ಕನ್ನಡ, മലയാളം, සිංහල, ไทย, ဖၠုံလိက်, ဘာသာ မန်, မြန်မာဘာသာ, ქართული, ትግርኛ, አማርኛ, ᐃᓄᒃᑎᑐᑦ / inuktitut, ភាសាខ្មែរ, 中文, 吴语, 日本語, 粵語, 閩南語, ꯃꯤꯇꯩ ꯂꯣꯟ, 한국어

### Tip 5: Improve existing data

The easiest first edit to make is to add a new statement to an existing item. Just use the button and Wikidata will attempt to autocomplete and suggest potential properties and items as you type. A good way to get started with editing is to check out the external identifiers section on an item’s page and perhaps add some missing identifiers for the concept from a database you are familiar with. So for example, if you are on an item about a taxon, you could check whether it correctly states the corresponding *GBIF taxon ID (P846)*, *NCBI taxonomy ID (P685)*, *MycoBank taxon name ID (P962)*, *IPNI plant ID (P961)*, *WoRMS-ID for taxa (P850)*, etc. These sorts of links out to external identifiers make Wikidata a valuable tool for easily cross referencing items between different resources for each concept.

Another good way to get started is to explore items about research articles and review—and possibly add—statements for *main subject (P921)*. A way of annotating such articles that is particularly unique to Wikidata is adding statements for *describes a project that uses (P4510)* to add important tools, techniques, or materials that the article highlights in its methods section. You can introduce a lot of extra richness to a statement including qualifiers via (Fig 1). The web interface can be customised with a range of extra tools and gadgets via your preferences to align its capabilities to what is most useful to you.

You can also edit an item’s short description using the button at the top. Even though these aren’t machine-readable, the text is useful for humans to disambiguate between items at a glance (for example, the word “translation” might indicate “the creation of proteins using information from nucleic acids” or “a function that moves every point a constant distance in a specified direction in euclidean geometry” or “transfer of meaning from one language into another”).

### Tip 6: Be bold, but not reckless

Like editing Wikipedia [1], the apparent complexity of Wikidata can make getting started seem intimidating. The trick is to start small. Try looking up Wikidata items on some key papers in your field of research (or this list of *PLOS Comp Biol* articles) and see if you can add its keywords as *main subject (P921)* or its methods as *describes a project that uses (P4510)*. Such annotation can get pretty detailed and granular as you can see in this example.

To work out how to best model new data you want to integrate, you can check out the showcases that many Wikiprojects maintain (see Tip 4) to see how similar item types should be organised for consistency. If your planned additions extend on current examples, involving those experienced contributor communities in the data modelling decisions can ensure that new content is modelled consistently with existing statements.

Remember, you can easily revert edits if you’ve made a mistake—go to the history tab at the top and click “undo.” If doing mass edits or additions (see Tip 9), remember to validate the updated data to make sure you’ve made the changes you intended to [8,23].

### Tip 7: Add references (cite, cite, cite)

**Fig 3:**
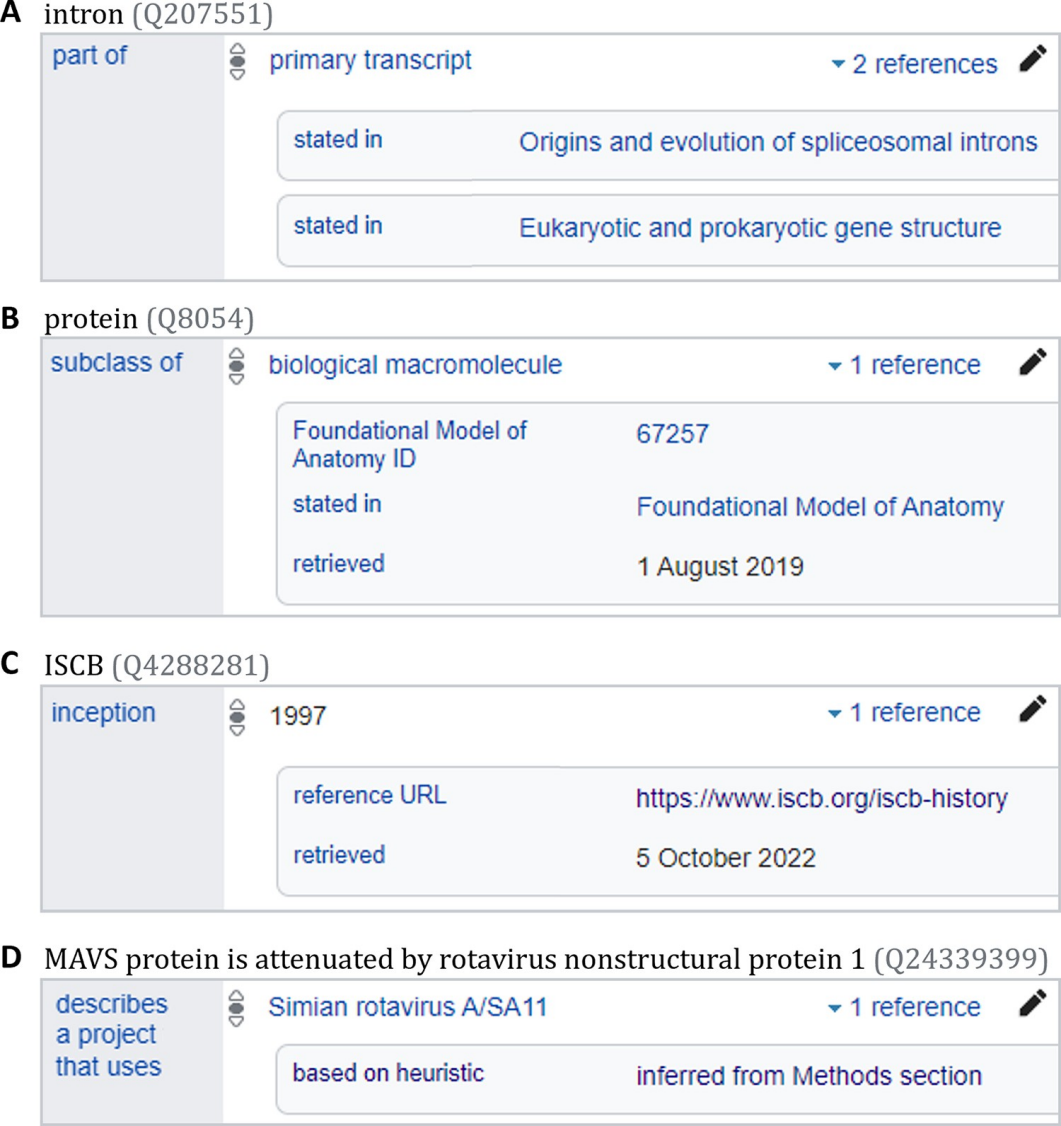
Example reference types to support statements. Examples of (**A**) referencing to Wikidata items for journal review articles, (**B**) referencing to a database entry, (**C**) referencing to a website, or (**D**) using a heuristic estimate to justify a statement. *These screenshots contain only text and data released under a CC0 licence*.

Just like in Wikipedia, Wikidata is primarily a secondary resource and acts as a hub or proxy to other resources, ideally in a way that facilitates verifiability. All statements should therefore, whenever possible, cite their provenance to existing knowledge in other external reliable sources. These are added via the button. To cite research articles, books, and other common reference types, you can reference their Wikidata QID (Fig 3A). If the source you want to use as a reference doesn’t have a Wikidata item yet, you can add it using tools such as Scholia. It is also possible to reference entries in external databases (Fig 3B) or webpages (Fig 3C). For sources that might change over time like databases and webpages—it is best to include the date retrieved or even an archived URL. Lastly, especially when a concrete reference isn’t possible, it is useful to provide the heuristic used (Fig 3D; list). It’s worth including citations for even seemingly trivial statements if a reference is available, for example, the statement that an *intron (Q207551)* is *part of (P361)* a *primary transcript (Q7243183)* references 2 papers (Fig 3A).

### Tip 8: Create new entities

Don’t be afraid to create new items. In general, each item should describe a single concept. For example, there are separate items for the *ɑ-defensin protein domain (Q4063641)*, *ɑ-defensin propeptide domain (Q24727071)*, *ɑ-defensin gene family (Q81639709)*, *ɑ-defensin 1 mouse gene (Q18248700)*, *ɑ-defensin 1 mouse protein (Q21421153)*, etc.

**Fig 4:**
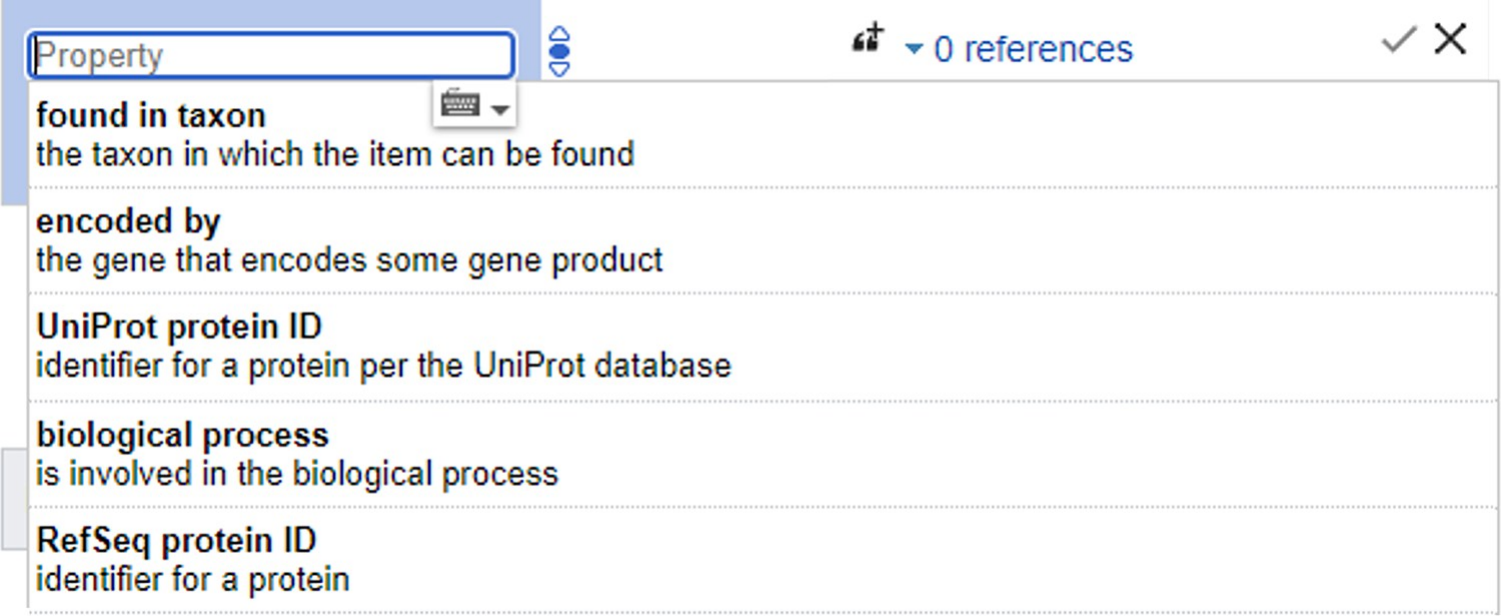
Property auto–suggestion. Once you start to add statements to an item (especially instance of/subclass of), the interface will begin to suggest common properties to add that other similar items include. Depicted are the suggestions given for a protein domain. For some properties, it will then also suggest common values for that statement. *This screenshot contains only text and data released under a CC0 licence*.

It is trivially simple to create a new item: the “create new item” link on the left will allow you to define an item, assign a short description, and add any aliases that it might also be known by. Newly created items always need to be given an *instance of (P31)* or *subclass of (P279)* statement to link it into the wider knowledgebase, but otherwise there are no compulsory fields. An easy way to identify additional statements to add is by checking items of a similar type. The interface will also attempt to suggest potential properties as you add statements (Fig 4). Although it’s best to avoid duplicates, merging items later is easy if it turns out there’s more than one for the same thing. You can also use Cradle where you can populate new items via a lightweight form which prompts you to include the most common fields.

While all scientific concepts fit on Wikidata in principle, there are notability guidelines that advise on which things should or should not have items. For example, valid taxa, type specimens, or reference genomes are essentially automatically notable. In contrast, not all humans are sufficiently notable, though researchers who have published peer-reviewed articles usually are.

Proposing new properties that can be used to link items is trickier. Compared to the >100M items, there are only 8K properties, so these have more a role of a controlled vocabulary. To propose a new property, simply list it and some example use cases at Wikidata:Property_proposal and experienced contributors will check if it makes sense to implement as proposed or with some changes or whether an already existing property can be adapted.

### Tip 9: Edit information in bulk

Once you’ve learnt how to add single statements and create single items, you’ll likely want to scale this up to edit information in bulk. Databases with a CC0 are becoming more common and can be integrated into Wikidata in full (e.g., CIViC, Wikipathways, Disease Ontology, and the Evidence and Conclusion Ontology). Other datasets (e.g., Uniprot, CC BY 4.0 licence) can still be integrated by linking out to them via external identifiers (example) or have their data integrated as a statement with proper referencing to attribute it (example).

When getting into larger scale editing, it is generally best to scale up test sets to identify any issues that come up—do a batch of 10 or a hundred edits before trying a thousand or a million. There are a range of ways to achieve this. There are Wikidata Tools available that cover a range of common situations. Editing tools can generally only be used after a minimum number of manual edits (typically 50) or a minimum age of the account (typically 4 days).

OpenRefine and Ontotext Refine take a spreadsheet of statements to be added and reconcile text strings in that spreadsheet to their most likely Wikidata items, flagging required manual intervention for ambiguous matches [24]. Ontotext Refine also contains an “RDF mapper,” which can help integrate Wikidata into external databases by generating a separate RDF that uses Wikidata’s identifiers but can be used outside of Wikidata. Quickstatements is a similar Wikidata editing tool, though it does not include the reconciliation functions so you’ll need to know any Wikidata QIDs to be included in statements beforehand [25]. Libraries are available in a range of languages (Table 1) to interface with Wikidata via its dynamic API and the query service. For example, the Wikidata integrator library can update items based on external resources and then confirm data consistency via SPARQL queries. It is used by multiple python bots to keep biology topics up to date, such as genes, diseases, and drugs (ProteinBoxBot) [14], or cell lines (CellosaurusBot) [26].

**Table 1:** Example Wikidata packages and libraries (extended list).

Language	Package/library
Python	Wikidata integrator or Pywikibot
Java	Wikidata toolkit
Javascript	WikbaseCLI.js
R	WikidataR

Since Wikidata is expressed as RDF, it comes with an EntitySchema extension [27] that enables describing the schema of captured knowledge as Shape Expressions (ShEx)—a formal language to describe data on the Semantic Web [28]. EntitySchema have been created for a range of item classes (list), for example, the *Protein Reactome Schema (E39)* or *clinical trial schema (E189)*. They act as documentation for the data deposited by data donors, but they also act as a document to describe expectations by users [8,28].

### Tip 10: Mind the gaps: What data is currently missing?

Wikidata is a secondary source for data and so, though it is rapidly growing, it will never be complete. This means that some level of inconsistency and incompleteness in its contents is currently inevitable [8,29–31]. There is thorough coverage of some items, such as protein classes [32], human genes [14], cell types [26,33], and metabolic pathways [34]. However, this is not true across all topics, and inconsistencies fall into a few categories (Box 2).

**Box 2. Main classes of data inconsistency in Wikidata**

(A) **Item incompleteness.** Since Wikidata is still in an exponential growth phase, it can be difficult to predict which topics will have already been well developed by the community and which are not yet well covered or linked out to external databases. For example, at time of writing, many common bioinformatics techniques and equipment types are currently missing. The opposite issue can also come up—duplicate items—which are resolvable via the merge function.
(B) **Statement incompleteness.** The issue of incompleteness can affect any part of Wikidata’s data model. For example, for many items about people, there are no statements about their date or place of birth. In cases where multiple statements are common for a given property on a given item (e.g., someone’s employer), some or all of them might be missing or outdated. Present statements can also sometimes be inconsistent—e.g., an occupation statement might include values that aren’t occupations, but rather a field of work, a produced good, or a genre.
(C) **Language incompleteness**. The language coverage for item labels and descriptions also varies, where core items—e.g., *evolution (Q1063)*—will be in hundreds of languages, whereas items towards the edge of the network—e.g., *evolvability (Q909622)*—may be in only a few languages or even just one.
(D) **Referencing incompleteness**. For example, at time of writing: The fact that the *SARS–CoV–2 NSP9 complex (Q89792653)* is found in SARS–CoV–2 is referenced (to the EBI complex portal), but that it contains 2 NSP9 subunits isn’t referenced.
(E) **Classification and description disparity**. For example, at time of writing, *principal component analysis (Q2873)* is listed as a subclass of multivariate statistics, used for dimensionality reduction, but *factor analysis (Q726474)* is listed as a subclass of statistical method, used for looking for latent variables. Some inconsistency also stems from the fundamental lack of a single universal classification (“how many countries exist?” being a classic example).
(F) **External databases and controlled vocabularies**. Mapping to external databases can vary, since some are proprietary or have other licensing issues, while others are simply incomplete. For example, there is currently minimal mapping over to Research Resource Identifiers, although a dedicated property for them exists—*Research Resource Identifier (P9712)*.

The practical upshot of this affects both contributing and using data. For adding new entities and new links between them, there is plenty to be done and huge scope for contribution. For using the data, it requires initial checking to ensure that it’s being stored in the structure that you’d expect (e.g., is the item listed as an instance of a gene, or instance of a protein, and for which species?) and that there aren’t obvious false negatives. Despite this, it is surprising how powerful Wikidata already is even in these early days, supporting COVID dashboards [7], a literature search engine [21], and genome browsers for several organisms [35].

## References

[1] Logan DW, Sandal M, Gardner PP, Manske M, Bateman A. Ten Simple Rules for Editing Wikipedia. PLoS Comput Biol. 2010;6:e1000941. doi: 10.1371/journal.pcbi.1000941 20941386 PMC2947980

[2] Waagmeester A, Stupp G, Burgstaller–Muehlbacher S, Good BM, Griffith M, Griffith OL, et al. Wikidata as a knowledge graph for the life sciences. Rodgers P, Mungall C, editors. Elife. 2020;9:e52614. doi: 10.7554/eLife.52614 32180547 PMC7077981

[3] Shafee T, Mietchen D, Su AI. Academics can help shape Wikipedia. Science. 2017;357:557–558. doi: 10.1126/science.aao0462 28798122

[4] Jemielniak D. Wikipedia: Why is the common knowledge resource still neglected by academics? GigaScience. 2019;8:giz139. doi: 10.1093/gigascience/giz139 31794014 PMC6889752

[5] Mora–Cantallops M, Sánchez–Alonso S, García–Barriocanal E. A systematic literature review on Wikidata. Data Technol Appl. 2019;53:250–268. doi: 10.1108/DTA–12–2018–0110

[6] Turki H, Shafee T, Hadj Taieb MA, Ben Aouicha M, Vrandečić D, Das D, et al. Wikidata: A large–scale collaborative ontological medical database. J Biomed Inform. 2019;99:103292. doi: 10.1016/j.jbi.2019.103292 31557529

[7] Turki H, Hadj Taieb MA, Shafee T, Lubiana T, Jemielniak D, Aouicha MB, et al. Representing COVID–19 information in collaborative knowledge graphs: The case of Wikidata. Semantic Web. 2022;13:233–264. doi: 10.3233/SW–210444

[8] Turki H, Jemielniak D, Hadj Taieb MA, Labra Gayo JE, Ben Aouicha M, Banat M, et al. Using logical constraints to validate statistical information about disease outbreaks in collaborative knowledge graphs: the case of COVID–19 epidemiology in Wikidata. PeerJ Comput Sci. 2022;8:e1085. doi: 10.7717/peerj-cs.1085 36262159 PMC9575845

[9] Waagmeester A, Willighagen EL, Su AI, Kutmon M, Gayo JEL, Fernández–Álvarez D, et al. A protocol for adding knowledge to Wikidata: aligning resources on human coronaviruses. BMC Biol. 2021;19:1–14. 33482803 10.1186/s12915-020-00940-y PMC7820539

[10] Dnshitobu MK, Abah A, Udeh B, Waagmeester A. Using Crowd–sourcing Platforms to Increase and Spread Knowledge on the Biodiversity in Sub–Saharan Africa. Biodivers Inf Sci Stand. 2022;6:e93803.

[11] Zárate M, Buckle C. LOBD: linked data dashboard for marine biodiversity. Springer; 2021. p. 151–164.

[12] Kilpatrick AM, Anjum A, Welch L. Ten simple rules for designing learning experiences that involve enhancing computational biology Wikipedia articles. PLoS Comput Biol. 2020;16:e1007868. doi: 10.1371/journal.pcbi.1007868 32407308 PMC7224448

[13] Meyer–Heß A, Rupp J. What happens if you publish the National Bibliography under a CC0 license?–Experiences of the German National Library (DNB). 2016. Available from: http://library.ifla.org/id/eprint/1414/.

[14] Burgstaller–Muehlbacher S, Waagmeester A, Mitraka E, Turner J, Putman T, Leong J, et al. Wikidata as a semantic framework for the Gene Wiki initiative. Database. 2016;baw015. doi: 10.1093/database/baw015 26989148 PMC4795929

[15] Erxleben F, Günther M, Krötzsch M, Mendez J, Vrandečić D. Introducing Wikidata to the Linked Data Web. In: Mika P, Tudorache T, Bernstein A, Welty C, Knoblock C, Vrandečić D, et al., editors. The Semantic Web–ISWC 2014. Cham: Springer International Publishing; 2014. p. 50–65. doi: 10.1007/978–3–319–11964–9_4

[16] Malyshev S, Krötzsch M, González L, Gonsior J, Bielefeldt A. Getting the Most Out of Wikidata: Semantic Technology Usage in Wikipedia’s Knowledge Graph. In: Vrandečić D, Bontcheva K, Suárez–Figueroa MC, Presutti V, Celino I, Sabou M, et al., editors. The Semantic Web–ISWC 2018. Cham: Springer International Publishing; 2018. p. 376–394. doi: 10.1007/978–3–030–00668–6_23

[17] Fichtmüller D, Reimeier F, Güntsch A. Using Wikibase as a Platform to Develop a Semantic TDWG Standard. Biodivers Inf Sci Stand. 2019;3:e37212. doi: 10.3897/biss.3.37212

[18] Shimizu C, Eells A, Gonzalez S, Zhou L, Hitzler P, Sheill A, et al. Ontology Design Facilitating Wikibase Integration––and a Worked Example for Historical Data. arXiv. 2022. doi: 10.48550/arXiv.2205.14032

[19] Rossenova L. Examining Wikidata and Wikibase in the context of research data management applications. In: TIB–Blog [Internet]. 16 Mar 2022 [cited 2023 Apr 21]. Available from: https://blogs.tib.eu/wp/tib/2022/03/16/examining–wikidata–and–wikibase–in–the–context–of–research–data–management–applications/.

[20] Dashnow H, Lonsdale A, Bourne PE. Ten Simple Rules for Writing a PLOS Ten Simple Rules Article. PLoS Comput Biol. 2014;10:e1003858. doi: 10.1371/journal.pcbi.1003858 25340653 PMC4207461

[21] Nielsen FÅ, Mietchen D, Willighagen E. Scholia, Scientometrics and Wikidata. In: Blomqvist E, Hose K, Paulheim H, Ławrynowicz A, Ciravegna F, Hartig O, editors. The Semantic Web: ESWC 2017 Satellite Events. Cham: Springer International Publishing; 2017. p. 237–259.

[22] Jemielniak D, Rychwalska A, Talaga S, Ziembowicz K. Wikiproject Tropical Cyclones: The most successful crowd–sourced knowledge project with near real–time coverage of extreme weather phenomena. Weather Clim Extremes. 2021;33:100354.

[23] Shenoy K, Ilievski F, Garijo D, Schwabe D, Szekely P. A study of the quality of Wikidata. J Web Semant. 2022;72:100679. doi: 10.1016/j.websem.2021.100679

[24] Delpeuch A. Running a reconciliation service for Wikidata. Proceedings of the 1st Wikidata Workshop. 2020. 17. Available from: http://ceur-ws.org/Vol-2773/paper-17.pdf.

[25] Aycock M, Critchley N, Scott A. Gateway into Linked Data: Breaking Silos with Wikidata. Texas Conference on Digital Libraries. 2021. Available from: https://digital.library.txstate.edu/handle/10877/13529.

[26] Lubiana T. Building a biological knowledge graph via Wikidata with a focus on the Human Cell Atlas. Manubot. Manubot; 2022 Jan. Available from: https://lubianat.github.io/quali_phd/.

[27] Extension:EntitySchema–MediaWiki. Available from: https://www.mediawiki.org/wiki/Extension:EntitySchema.

[28] Thornton K, Solbrig H, Stupp GS, Labra Gayo JE, Mietchen D, Prud’hommeaux E, et al. Using Shape Expressions (ShEx) to Share RDF Data Models and to Guide Curation with Rigorous Validation. In: Hitzler P, Fernández M, Janowicz K, Zaveri A, Gray AJG, Lopez V, et al., editors. The Semantic Web. Cham: Springer International Publishing; 2019. p. 606–620. doi: 10.1007/978–3–030–21348–0_39

[29] Strickler CM. Mind the Wikidata Gap: Why You Should Care About Theological Data Gaps in Wikipedia’s Obscure Relative, and How You Can Do Something About It. Atla Summary of Proceedings. 2021:301–314. doi: 10.31046/proceedings.2021.2978

[30] Piscopo A, Simperl E. What we talk about when we talk about wikidata quality: a literature survey. Proceedings of the 15th International Symposium on Open Collaboration. New York, NY, USA: Association for Computing Machinery; 2019. p. 1–11. doi: 10.1145/3306446.3340822

[31] Amaral G, Piscopo A, Kaffee L, Rodrigues O, Simperl E. Assessing the Quality of Sources in Wikidata Across Languages: A Hybrid Approach. J Data Inf Quality. 2021;13(23):1–23:35. doi: 10.1145/3484828

[32] User:ProteinBoxBot–Wikidata. [cited 2022 Oct 5]. Available from: https://www.wikidata.org/wiki/User:ProteinBoxBot.

[33] Lubiana T. Cell Type Query Book. 2022. Available from: https://github.com/lubianat/cell_type_query_book.

[34] Martens M, Ammar A, Riutta A, Waagmeester A, Slenter DN, Hanspers K, et al. WikiPathways: connecting communities. Nucleic Acids Res. 2021;49:D613–D621. doi: 10.1093/nar/gkaa1024 33211851 PMC7779061

[35] Putman TE, Lelong S, Burgstaller–Muehlbacher S, Waagmeester A, Diesh C, Dunn N, et al. WikiGenomes: an open web application for community consumption and curation of gene annotation data in Wikidata. Database. 2017:bax025. doi: 10.1093/database/bax025 28365742 PMC5467579

